# Japanese encephalitis virus tropism in experimentally infected pigs

**DOI:** 10.1186/s13567-016-0319-z

**Published:** 2016-02-24

**Authors:** Meret E. Ricklin, Obdulio Garcìa-Nicolàs, Daniel Brechbühl, Sylvie Python, Beatrice Zumkehr, Horst Posthaus, Anna Oevermann, Artur Summerfield

**Affiliations:** Institute of Virology and Immunology, Mittelhäusern, Switzerland; Vetsuisse Faculty, Institute for Animal Pathology, University of Bern, Bern, Switzerland; Division of Neurological Sciences, DCR-VPH, Vetsuisse Faculty, University of Bern, Bern, Switzerland; Department of Infectious Diseases and Pathobiology, Vetsuisse Faculty, University of Bern, Bern, Switzerland

## Abstract

Pigs are considered to be the main amplifying host for Japanese encephalitis virus (JEV), and their infection can correlate with human cases of disease. Despite their importance in the ecology of the virus as it relates to human cases of encephalitis, the pathogenesis of JEV in pigs remains obscure. In the present study, the localization and kinetics of virus replication were investigated in various tissues after experimental intravenous infection of pigs. The data demonstrate a rapid and broad spreading of the virus to the central nervous system (CNS) and various other organs. A particular tropism of JEV in pigs not only to the CNS but also for secondary lymphoid tissue, in particular the tonsils with the overall highest viral loads, was observed. In this organ, even 11 days post infection, the latest time point of the experiment, no apparent decrease in viral RNA loads and live virus was found despite the presence of a neutralizing antibody response. This was also well beyond the clinical and viremic phase. These results are of significance for the pathogenesis of JEV, and call for further experimental studies focusing on the cellular source and duration of virus replication in pigs.

## Introduction

Japanese encephalitis virus (JEV) causes an important zoonotic, vector-borne disease present in East Asia, Southeast Asia and Australasia [1, 2]. During JEV epidemics, human infections are widespread but only 0.1–4% of infected individuals develop clinically apparent encephalitis. In the past, the annual incidence of such human cases was in the range from 50 000 to 175 000 [1–3]. Importantly, the mortality of encephalitis cases is as high as 25–30%, and approximately 50% of the surviving patients suffer from neuro-psychiatric sequelae [1, 2]. Therefore, JEV is considered to be the most frequent viral encephalitis associated with fatal outcomes [4]. JEV is currently endemic in large parts of Southeast Asia but similar to other emerging diseases, globalization and climate changes may result in virus emergence in the Western hemisphere. This would cause significant health problems in both the naïve human and animal population. During the last two decades West Nile virus as well as Usutu virus, which both belong to the JEV serocomplex, emerged in Europe, demonstrating that related mosquito-born *flaviviruses* can spread to and remain in temperate regions [5]. Furthermore, the temperate northern Island of Japan Hokkaido, was affected by JEV epidemics and the virus was shown to overwinter and re-emerge in the same local region [6].

The ecology of JEV includes a bird-associated wildlife cycle between *Culex* mosquitoes as vectors and mostly water birds such as egrets and herons as reservoirs. However, it appears that a high level of JE incidence in humans is associated with the presence of pigs as amplifying hosts [1, 2]. Already early studies in the 1950s have demonstrated that pigs are readily infected and develop high level of viremia for several days [7–9]. This fact is an important feature of a pig-associated rural cycle, present mostly in areas with intense pig farming and rice fields, which have been associated with frequent human JE cases [10].

While clinical symptoms in pigs are mild, humans and horses develop severe disease, and are considered as dead end hosts in which viremia is insufficient to infect feeding mosquitos [1]. High seroprevalence in many other vertebrates, such as dogs, chickens, ducks and reptiles indicate widespread infection by the virus but their role in the ecology of the virus is unclear [1]. Certainly, the high propensity of *Culex tritaeniorhynchus* to feed on pigs and the high birth rate with rapid turnover of the pig population favor the hypothesis of the pig as being the main amplifying host for JEV leading to human JE cases [1, 2].

During natural infection of pigs in JEV endemic areas, a viremic phase lasting 2–4 days has been described [7, 9, 11]. Clinically, JEV field infections have been associated with increased rates of stillbirth and abortion [6, 12, 13]. Experimental infection of pigs resulted in development of transient fever and depression associated with a non-suppurative encephalitis with viral antigen distributed broadly within the central nervous system (CNS) [14, 15].

Despite this information, the pathogenesis of JEV in pigs remains obscure. Specifically, the localization and kinetics of virus replication associated with viremia have not been studied. Furthermore, data of pig breeds kept in Europe such as Landrace, Large White or Duroc are lacking. The present study demonstrates that after experimental infection of pigs JEV has a strong tropism not only to the CNS but also for secondary lymphoid tissue, in particular the tonsils.

## Materials and methods

### Animal experiment

All animal experiments were performed at biosafety level 3 conditions (BSL3), and under the guidance of the Swiss animal welfare law, approved by the Cantonal ethical committee for animal experiments (BE 118-13). Twelve healthy seven-weeks old swiss large white pigs (seven castrated males and five females) from our specific-pathogen-free (SPF)-breeding facility were used. Animals were housed in groups of ≥3 inside the containment facility of the Institute for Virology and Immunology (IVI) representing a BSL3-Ag facility. Prior to the inoculation experiment, they were allowed 1 week of adaptation to the new environment. After puncturing the jugular vein for a null blood sample all 12 animals were infected with 10^7^ TCID_50_ of JEV (Nakayama strain, obtained from the National collection of pathogenic viruses, NCPV, Salisbury, UK) in a volume of 2 mL, giving one half of the dose intravenously and the other half intradermally in the neck region. The Nakayama strain represents human genotype III isolate. It was used after two passages on Vero cells (ATCC, Manassas, VA, USA). Considering the low number of pigs possible for this study a high dose was chosen to ensure infection of all animals.

Animals were clinically examined daily, which included measuring body temperature and assessing awareness, appetite, manure excretion, breathing, gait and neurological signs. Daily blood samples and oro-nasal swabs (Sarstedt, Nümbrecht, Germany) were collected from three animals until day 11. Swab samples were drenched in 500 µL medium and the stored at −70 °C until further processing.

To collect organ samples, groups of three pigs were euthanized by electroshock and subsequent exsanguination at days 3, 5, 7 and 11 post infection (pi). Sampling was performed immediately after exsanguination and included collection of blood (for serum and isolation of leukocytes) and organs for quantitative reverse transcriptase polymerase chain reaction (qRT-PCR), virus isolation and histology. The following organs were sampled: cervical lymph node, tonsils, spleen, ileum, bone marrow, thymus, kidney, liver, skeletal muscle and a peripheral nerve (nervus auriculopalpebralis) extracted from the neck region used for intradermal infection. The brain, spinal cord and spinal ganglia were taken out in toto. Of the CNS, the following parts were collected: cervical and sacral spinal cord, brain stem, cerebellum, thalamus, hippocampus, basal nuclei, neocortex frontalis and temporalis, bulbus olfactorius, the meninges and choroid plexus. For histology organ samples were fixed in 4% buffered formalin for 3 days or 4 weeks for brain tissue. Peripheral blood mononuclear cells (PBMC) were isolated by density gradient centrifugation as previously described [16].

### Virological analyses

Organ samples were taken into 1.5 mL tubes (Sarstedt) containing 500 µL MEM medium (Gibco, Life Technologies, Zug, Switzerland) and were weighed before homogenizing with a BulletBlender^®^ (Next Advanced Inc. Averill Park, NY, USA). Homogenized organs were centrifuged and the supernatant transferred into a new tube to freeze immediately at −70 °C. For qRT-PCR, the samples were thawed and spiked with EGFP RNA as internal control prepared as described [17], and RNA was extracted using the QIAmp^®^ viral RNA extraction kit (Qiagen AG, Hombrechtikon, Switzerland) according to manufacturer’s instructions. The real-time RT-PCR was performed as published previously [18], using the SuperScript^®^ III Platinum^®^ One-Step qRT-PCR Kit (Life Technologies) and run on a 7900HT Thermocycler (Applied Biosystems) for 50 cycles. Results were used only if the EGFP RT-PCR showed a CT value <28 to assure quality of the RNA extraction and PCR reaction. To relatively quantify viral load, RNA obtained from a serially diluted stock of JEV Nakayama with a known titer was used as a standard. To this end, a virus stock with a known infectivity titer was serially diluted in log_10_ steps and viral RNA extracted. After qRT-PCR, CT values were determined to draw a standard curve, which was linear (correlation coefficient R of 0.998) in the range of 35.6–12.6 CT which corresponded to 1 × 10^0^–1.2 × 10^7^ TCID_50_/mL of the viral stock. The CT value corresponding to 1 TCID_50_ was defined as 1 RNA unit (U). Using this standard, the CT values of our samples were then transformed into relative quantities as RNA U/mL. Organ samples were corrected for their weight and data calculated as relative RNA quantities/mg.

To determine infectious virus titers, samples were serially diluted in quadruplicates starting at a dilution of 1:2 (if possible), and 100 μL was added to confluent Vero cells (ATCC, Manassas, VA, USA) cultured in 96-well plates with MEM medium (Life Technologies) containing 1% fetal bovine serum (FBS; Biochrome) and 0.01 M HEPES (Life Technologies). After 4 h, the inoculum was removed, replaced with fresh medium and the cells were incubated for 72 h before fixing them with 4% paraformaldehyde (Polysciences, Warrington, PA, USA) for 10 min and staining with the anti-flavivirus E protein monoclonal antibody 4G2 (HB-112™ ATCC) in saponin (Sigma-Aldrich Chemie GmbH, Buchs, Switzerland) followed by horseradish peroxidase conjugated rabbit anti-mouse antibody (Dako, Baar, Switzerland). The final color reaction was obtained by adding 3-amino-9-ethylcarbazole (AEC) (Sigma-Aldrich). Titers were calculated using the Reed and Munch formula. The theoretical detection limit of the virus titration was around 5 TCID_50_/mL.

### Histopathology

For histopathological evaluation organ samples and cross-sectioned blocks of representative areas of the CNS (medulla oblongata, pons, cerebellum, midbrain, thalamus, basal nuclei, olfactory bulb, hippocampus, cerebral cortex and spinal cord were embedded in paraffin, cut at 4 µm and stained with hematoxylin and eosin (HE).

Lesions in the CNS were semi-quantitatively scored from 0 to 4 (0 = no lesions, 1 = minimal lesions, 2 = mild lesions, 3 = moderate lesions, 4 = severe lesions). The scores were based on the number of lesions, the type including perivascular cuffs, neuronal necrosis, glial nodules and parenchymal infiltration by inflammatory cells, as well as size of lesions. Grade 1 was attributed to the presence of single thin perivascular cuffs only or focal small glial nodule. Grade 2 included few perivascular cuffs and few glial nodules. Grade 3 was characterized by prominent perivascular cuffs and multiple dense glial nodules with clear neuronal degeneration and neuronophagia (as exemplified in Figure 4). Grade 4 was characterized by extended lesions with prominent perivascular cuffs and numerous glial nodules, that may coalesce, and frequent evidence of neuronophagia. Three healthy, non-infected pigs from the IVI SPF facility were employed as negative controls.

### Antibody responses

For plaque reduction neutralization tests (PRNT) sera were diluted in medium in triplicates starting at 1:5 and twofold serially diluted to a dilution of 1:640. One hundred plaque forming units (PFU)/well of homologous virus were added to each well followed by gentle agitation and incubation at 37 °C for 30 min. Confluent Vero cells in flat bottom 96 well plates (Corning, Sigma Aldrich) were then incubated with 25 µL of the serum-virus mix for 1 h at 37 °C before washing with warm MEM medium (as above) and adding 200 µL of 1% methylcellulose (Sigma-Aldrich) medium supplemented with 100 IU penicillin and 100 µg/mL streptomycin at 200 µL/well. After incubation for 48 h at 37 °C, the cells were fixed and stained as described above. PRNT_50_ titres were read as the last serum dilution that showed a reduction of the counted PFU by 50%.

### Cytokine ELISAs

Serum prepared from blood samples was analyzed for interferon-α (IFN-α), interleukin-1β (IL-1β), tumor necrosis factor-α (TNF-α) and interleukin-6 (IL-6) using commercial porcine cytokine ELISA kits (R&D Systems, Abingdon, UK) according to manufacturer’s instructions.

### Statistical analysis

Statistical analysis was done with GraphPad Prism (GraphPad Software, La Jolla, USA) using ANOVA and Mann–Whitney U test to calculate group differences. Significance level was set at 5%.

## Results

### Clinical signs

**Figure 1:**
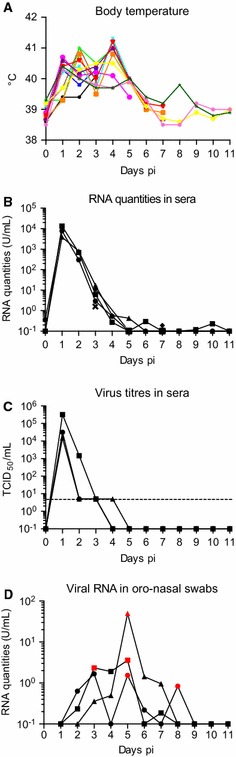
**Development of body temperature and viremia following experimental infection of pigs with JEV.** 12 piglets were infected with 10^7^ TCID_50_ JEV Nakayama strain. Pigs were killed in groups of three on days 3, 5, 7, and 11 pi. From three pigs daily serum samples were obtained, and from nine pigs blood samples were collected at necropsy (days 3, 5, 7 and 11 pi). **A** Daily body temperature of all animals. Each colour represents an individual animal. **B** Viral RNA loads in the serum. Relative JEV RNA quantities were determined using real-time RT-PCR. Quantification employed a standard curve created using a viral preparation with a known infectious titre. One U was defined as the viral RNA quantity corresponding to 1 TCID_50_ of the virus preparation used as standard. **C** Viral titres in the serum of individual bled daily. The detection limit of the assay was 5 TCID_50_/mL (dotted line). **D** Viral RNA loads in the oro-nasal swabs collected daily from three different animals. The red symbols represent samples from which live virus isolation succeeded.

Before experimental infection all 12 piglets were healthy and alert and had body temperatures of 38.7–38.9 °C. Already 24 h after infection all of them showed an increase in body temperature of up to 40.8 °C that lasted for 2–5 days before dropping to pre-infection levels (Figure 1A). Four pigs even reached body temperatures of above 41 °C. Fever curves were double peaked in most pigs. All animals showed reduced appetite, produced less manure and were reluctant to move for 3–6 days. With the normalization in body temperature also the clinical symptoms disappeared. However the three pigs kept until day 11 appeared to be depressed and showed a decreased appetite until day 9 pi.

### Viremia

The three pigs sampled for blood daily showed a prominent viremia in terms of viral RNA with a peak at day one pi and a viral RNA load of around 10 000 RNA U/mL (Figure 1B). Based on a standard curve performed, one RNA U corresponds to 1 TCID_50_ infectious virus. Viremia decreased gradually by 1–2 logs_10_ per day until day 4 pi. Sera from animals slaughtered on day 3 pi still showed a viremia above the threshold of the real-time RT-PCR with relative RNA quantities of 10 units/mL of serum. On day 5 all pigs were below cut-off of the RT-PCR. To determine how viral RNA levels correlate to live virus detection by cell culture we titrated some of the sera. As shown in Figure1C, a comparable kinetic was found with three representative sera from the animals bled daily. In contrast to the serum, no viral RNA was detected in PBMC.

### Oro-nasal excretion of JEV

Interestingly, we were able to detect viral RNA and live virus in oro-nasal swabs collected from the three animals which were kept until day 11. As shown in Figure 1D, this was found between 2 and 8 days pi, depending on the animal.

### Virus tropism for non-CNS tissue

**Figure 2:**
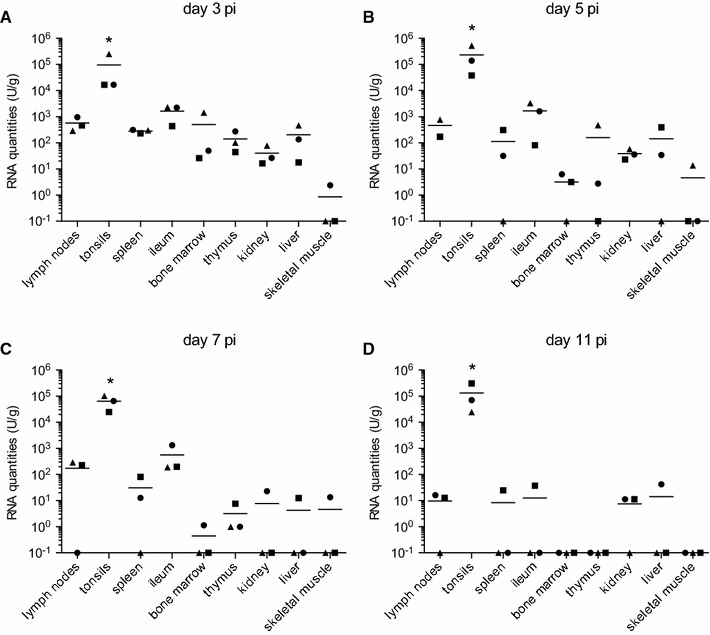
**Viral RNA loads in the peripheral organs following experimental infection of pigs with JEV.** At necropsy peripheral organs were sampled and the tissue homogenized before quantitative RT-PCR was run. Relative RNA quantities are shown for each day (**A**–**D**) calculated in U/mL as explained in the legend of Figure 1 and Materials and methods. Bars indicate mean values of a group. To illustrate RT-PCR negative samples they were plotted as 10^−1^ U/mL. *difference statistically significant (*p* < 0.05) when this organ is compared to all others for a particular time point pi.

At day 3 pi (Figure 2A), all sampled organs including lymph nodes, spleen, terminal ileum, bone marrow, thymus, kidney and liver were positive, mostly with viral RNA loads of 10–1000 RNA U/g. While the viral RNA load in the skeletal muscle was below 10 RNA U/g or negative, the tonsils contained 2–3 logs_10_ higher viral RNA loads reaching levels as high as 5 × 10^5^ RNA U/g. At the later time points (Figures 2B–D) a gradual decrease of the viral loads in all organs, except the tonsils, was observed. Due to inevitable blood contamination some of the organs might have false positive viral loads, although in the peripheral blood only around 10 RNA U/mL were measured at 3 days pi. Interestingly, even at 11 days pi (Figure 2D), there was no reduction in viral RNA loads in the tonsils. Also in the lymph node, spleen, terminal ileum (containing continuous Peyer’s patches in the pig), liver and kidney viral RNA was still found at day 11 pi in some animals. The high levels of viral RNA in the tonsils were associated with detection of live virus in all tonsils obtained from infected pigs until 11 days pi with viral loads of up to 3 × 10^5^ TCID_50_/g (Table 1).

**Table 1:** **Virus isolation data from tonsils**

Pig #	Days pi	Virus titration^a^ (TCID_50_/g)
1	3	3.14 × 10^4^
2	3	3.16 × 10^5^
3	3	3.16 × 10^5^
4	5	1.47 × 10^3^
5	5	3.16 × 10^5^
6	5	3.14 × 10^4^
7	7	3.16 × 10^5^
8	7	1.00 × 10^4^
9	7	1.78 × 10^4^
10	11	4.39 × 10^2^
11	11	6.81 × 10^4^
12	11	3.16 × 10^3^

^a^Theoretical sensitivity 5 × 10^1^ TCID_50_/g.

### Virus tropism for CNS tissue

**Figure 3:**
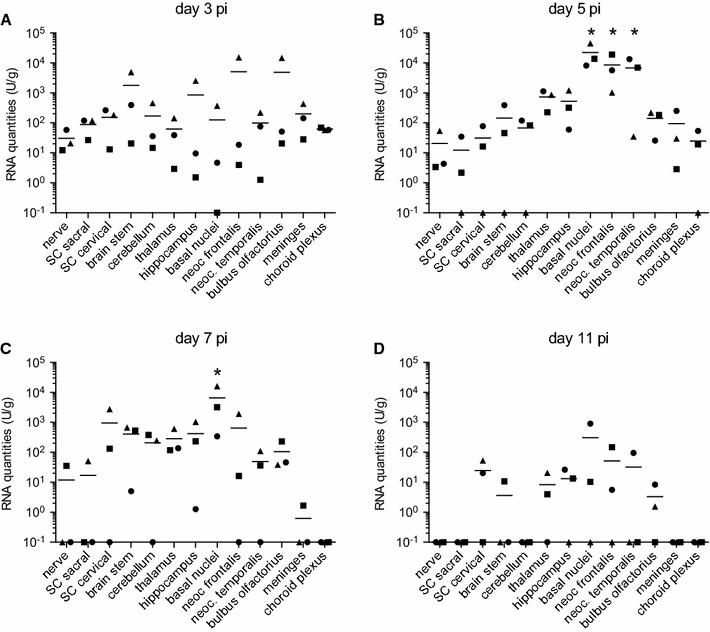
**Viral RNA loads in the nervous system following experimental infection of pigs with JEV.** At necropsy nervous tissues were sampled and homogenized before quantitative RT-PCR was run. Relative RNA quantities are shown for each day (**A**–**D**) calculated in U/mL. Bars indicate mean values of a group. To illustrate RT-PCR negative samples they were plotted as 10^−1^ U/mL. *difference statistically significant (*p* < 0.05) when this organ is compared to all others for a particular time point pi.

Even though no clinical neurological signs could be observed, most tested areas of the CNS contained viral RNA from day 3 to 7 pi (Figure 3). In some tissues such as thalamus, neocortex, basal nuclei a peak of viral RNA was observed at day 5 pi (Figure 3B). At day 11 pi, the end of the observational period, the CNS tissues of some animals did not contain detectable viral RNA anymore (Figure 3D). Peripheral nerve tissue, the spinal cord and meninges had relatively lower viral RNA levels, which decreased rapidly, whereas higher values were found in the basal nuclei and the neocortex (frontalis and temporalis). Meninges and the choroid plexus remained negative throughout the observation period.

### Pathology

**Figure 4:**
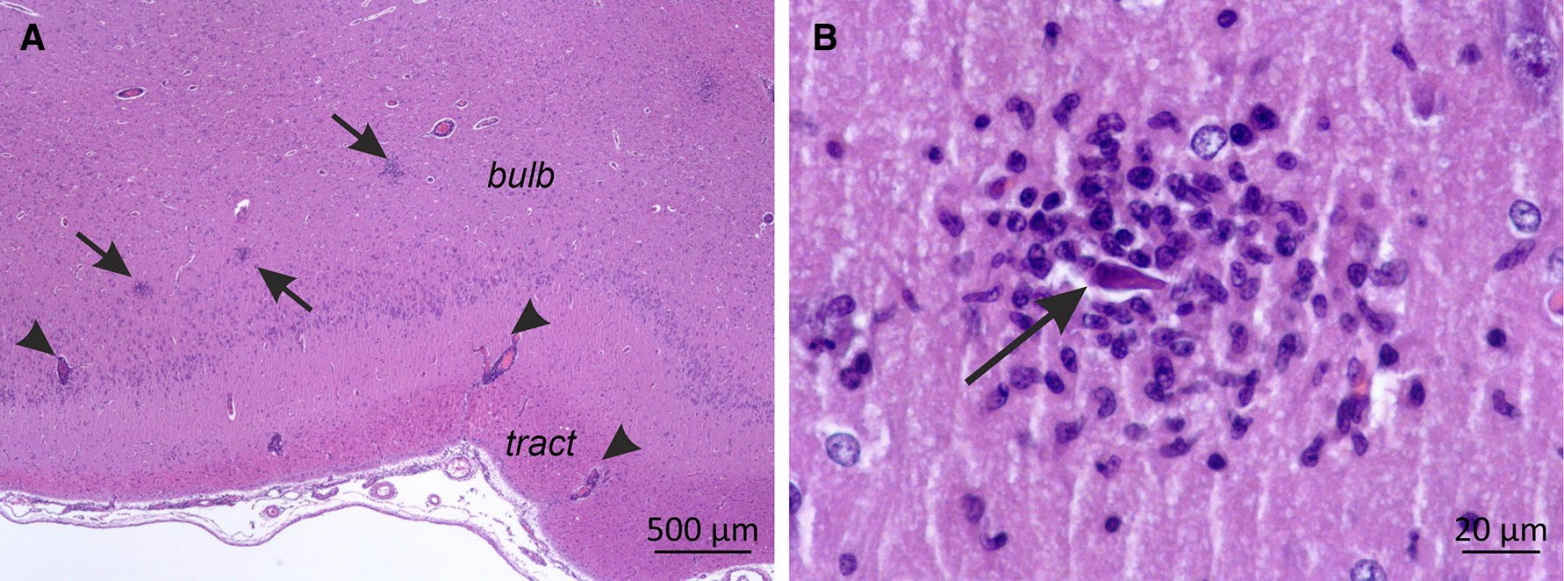
**Neuropathology in the olfactory bulb of a JEV-infected pig at 5** **days pi (score 3).** **A** Histopathology of the olfactory bulb (bub) and tract (tract). Multifocal glial nodules were present in the grey matter of the olfactory bulb (arrows) and accompanied by mononuclear perivascular cuffs (arrowheads). H and E, 20× magnification, bar = 500 µm. **B** Higher magnification (400×) of one glial nodule shown in **A**). One degenerated, shrunken neuron (arrow) in the olfactory bulb is surrounded by a nodule of microglial and inflammatory cells. H and E, bar = 20 µm.

**Figure 5:**
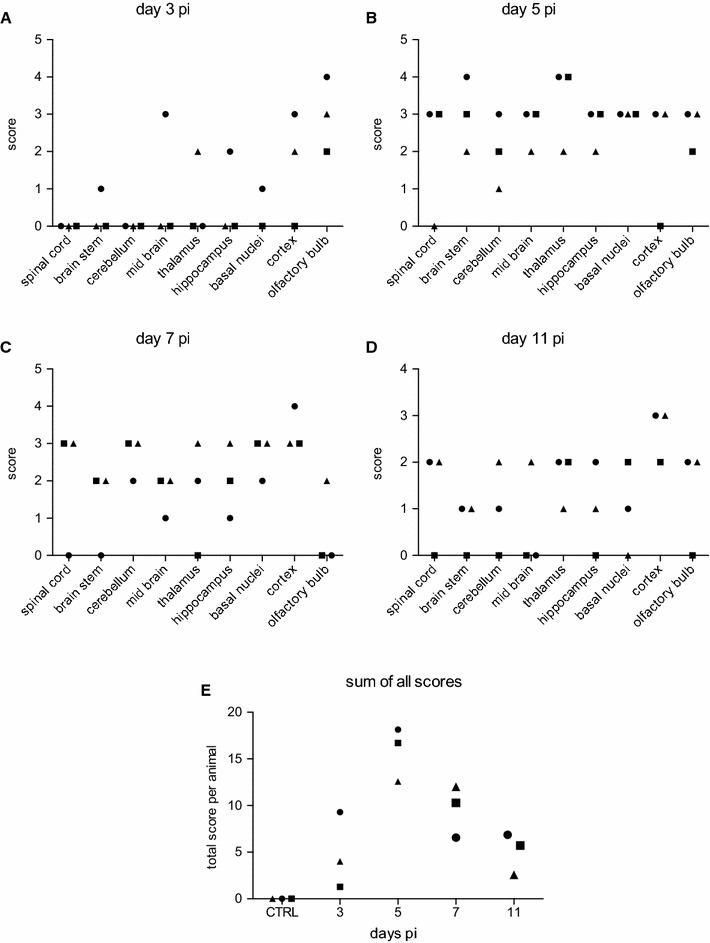
**Histological scores in the CNS following JEV-infection of pigs.** At necropsy brains and the spinal cord were sampled, fixed and processed routinely for H and E staining. Data are shown for each day (**A**–**D**) separately. Lesions were scored semi-quantitatively from 0 to 4 (0=no lesions, 1=minimal lesions, 2=mild lesions, 3=moderate lesions, 4*=*severe lesions). Scores of three healthy control piglets (CTRL) were 0. **E** Sum of all CNS tissues scores with each symbol representing an individual pig.

Macroscopically, in five of the 12 animals a slightly increased amount of abdominal fluid was present on days 5 and 7. These five animals were those with the more severe clinical signs. No other apparent macroscopic alterations were observed in any of the 12 animals. In the non-CNS tissues no histological changes were observed except signs of immune activation in secondary lymphoid tissue, particularly in the tonsils. In contrast, in the brain typical lesions of a viral meningoencephalomyelitis were observed. Lesions were characterized by multifocal lymphohistiocytic perivascular cuffs affecting mainly the grey matter and to a lesser degree the white matter, associated with the presence of glial nodules and evidence of neuronal degeneration and necrosis (Figure 4). Frequently, few neutrophils were present in the areas of neuronal necrosis. Additionally, multifocal mild lymphohistiocytic meningitis was present. The severity of lesions varied between animals, anatomical regions and over time. Lesions were mildest at day 3 pi (Figure 5A). The olfactory bulb was the only brain region clearly affected at this time point. Here, the lesions reached a peak between day 3 and 5 pi, whereas in all other CNS regions the most severe lesions were observed at day 5 or 7 pi (Figures 5B and C). When the scores allocated to the different areas of the CNS were added for each individual pig, the highest values were obtained at 5 days pi (Figure 5E).

### Immune responses

To determine if JEV induced an early systemic inflammatory response, serum obtained from the daily blood samples was analyzed by ELISA for IFN-α, IL-1β, IL-6 and TNF-α. In none of the samples cytokines levels above those of non-infected healthy pigs were found (data not shown).

**Figure 6:**
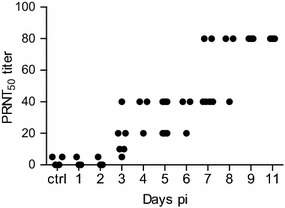
**Development of neutralizing antibodies following experimental infection of pigs with JEV.** All sera were tested for their neutralization ability using a standard plaque neutralization assay. Titres are calculated as the last serum dilution that reduces viral plaques by 50%.

All infected pigs rapidly seroconverted in terms of developing neutralizing antibodies. Already at 3 days pi two pigs showed a PRNT_50_ titer of 10 against the homologous virus which was above the non-specific PRNT_50_ titer of ≤5 found before infection. At day 5 all animals clearly seroconverted with increasing titers at 7 and 11 days pi (Figure 6).

## Discussion

Within the last two decades several vector borne viral diseases have emerged in Europe [19, 20]. The observation that JEV RNA was found in *Culex pipiens* in Italy represents a warning, although this was only described in one study [21]. In terms of mosquito vectors *Culex* and *Aedes* species, which can potentially transmit JEV, are present in southern Europe up to the south of Switzerland [22]. Considering that some areas in Europe have intensive pig farming, introduction of JEV could potentially result in severe health and socio-economical consequences. Pigs are considered to be the main amplifying host and important reservoirs along with water birds. In order to estimate the risk of a potential emergence of JEV into the Western hemisphere, it is important to know if local breeds of pigs are susceptible to viral infection, and in particular if they develop a viremia to a duration and magnitude comparable to Asian breeds of pigs. Our study indicates that this is the case at least with Swiss Large White pigs. The clinical signs observed were comparable to findings of previous reports with fever of between 40 and 41 °C for 3–5 days, with highest body temperatures beyond the end of the viremia. This was associated with decreased appetite, awareness and depression. The observed symptoms were possibly caused by the viral encephalitis and not by a virally induced systemic inflammation [14, 15]. With our infection model, viremia lasted for 2–4 days with the peak at day 1 pi. This was comparable to the study of Sasaki and colleagues in which the pigs were viremic from day 1 to 4 pi using mosquito bite and subcutaneous injection [23]. Other studies also found a viremia of 1–3 days duration occurring between day 1 and 5 pi [24, 25].

As in none of the previously published studies viral loads in various tissues were determined, the viral tropism of JEV in the CNS and non-CNS tissue of pigs required investigations. For this reason, we performed real-time RT-PCR in various tissues collected from serially slaughtered pigs. Our data indicate that in the bone marrow, thymus, kidney, liver and skeletal muscle, the virus is not present much longer than the viremia is lasting, although individual variations were observed. In contrast, in the brain, as well as in secondary lymphoid tissue including spleen, lymph node and in particular the tonsils virus was detectable clearly beyond the viremic phase. As for the ileum, which had comparable viral RNA loads to the lymph nodes, we collected the terminal part containing the continuous Peyer’s patches. We speculate that also in this organ the site of virus replication is in lymphoid tissue. Altogether, our data indicate that the virus has a relatively broad tissue tropism with a preference for nervous and lymphoid tissues. Nevertheless, although PBMC can be infected in vitro [26, 27], PBMC isolated from infected animals remained negative in the RT-PCR throughout the experiment. Future in vitro and in vivo studies are now required to determine the cellular tropism of JEV in pigs. Especially lymphoid tissue will be of large interest.

Although clinical signs were mild and no clear neurological deficits were observed, all animals were affected by non-suppurative meningoencephalomyelitis. Lesions in most brain regions appeared at day 3 and persisted until day 11 pi. The spinal cord and cerebellum were affected from day 5 on. Lesions and their distribution were similar to JEV-meningoencephalomyleitis previously described in experimental intravenous infections of pigs and macaques [14, 15, 28] and in natural infections of human [29–31]. Similar to these studies, we found neuronal degeneration and necrosis confirming a neurotropism of the JEV strain used [14, 15, 28]. Although the olfactory bulb appeared to be slightly stronger affected than other CNS regions at day 3, the widely disseminated distribution of lesions was compatible with a hematogenous virus entry into the CNS [14], possibly by free virus particles during the viremic phase as indicated by our serum RT-PCR results. The absence of JEV in PBMC indicate that these cells do not function as Trojan horses for JEV entry into the CNS as it has been described for other viruses [32, 33]. As described in mice JEV entry into the CNS via the blood-cerebrospinal fluid barrier appears to be unlikely as neither viral RNA or lesions were observed in the choroid plexus nor in ependymal or periventricular tissue [34]. Future studies also need to consider alternatives routes of viral entry into the CNS, such as transneural virus transport, which may occur for example from the olfactory nerve to the olfactory bulb from which the virus may further spread to the CNS as has been described for other neurotropic flaviviruses (for review see [35]). This may link virus replication in the tonsils with spread to the CNS.

The most striking novel observations in terms of JEV tropism were found in the tonsils, in which JEV appeared to continue its replication for at least 11 days. This was 6 days after the end of viremia and the onset of neutralizing antibody responses. In addition, viral loads were 100–1000 times higher compared to other organs. Only in one previous study, JEV was isolated from the tonsil of a pig [14] but to our knowledge, viral infection of the tonsils in pigs or other species has not been described. It should be noted that the present study employed a high dose of JEV (10^7^ TCID_50_/animal), which could theoretically result in a different tissue distribution as a natural infection. However, although we do not have samples from naturally infected pigs available, data from several pigs infected with doses of JEV as low as 10^3^ TCID_50_/animal via the oro-nasal route resulted in a comparable tissue distribution. Again, the highest viral loads are also found in the tonsils [36]. This suggests that although the kinetic of the infection and the incubation time may vary, the virus distribution reported here is relevant for the field situation.

Future studies are required to determine how long the virus can persist in pigs as a long-term persistence could have an important impact on the ecology of JEV in temperate regions. In fact, for recurrent JEV outbreaks observed in particular farms in consecutive years in Hokkaido, the mechanisms of hibernation of JEV remain unclear [6, 37]. Although experimentally JEV can transmit vertically in *Culex* mosquitoes [38, 39], it has been very difficult to find infected mosquito larvae in the field [40].

Previous studies indicate that neutralizing antibodies are important for protection against JEV with a protective neutralizing titer of 10, in both humans and in pigs [25, 41–43]. Our study confirms that the immune system of pigs efficiently and rapidly reacts to JEV infection. Within one weak pi, similar levels of neutralizing antibodies are reached as compared with a live attenuated vaccine [23] conferring protective immunity. Under our experimental conditions JEV did not induce systemic inflammatory cytokine response also supporting a rapid control of virus replication.

Taken together this study demonstrates that after experimental infection of pigs JEV rapidly and broadly spreads to the CNS and various organs outside the CNS. It appears that the secondary lymphoid tissue plays a prominent role in virus replication with the tonsils being a predilection site for JEV in terms of both viral loads and duration of replication. Considering the important role of pigs in the ecology of JEV, future investigations are required to understand virus-host interactions at the level of the organism and the cell.
